# *N*,*N*′-Bis(4-bromo­benzyl­idene)-2,2-dimethyl­propane-1,3-diamine

**DOI:** 10.1107/S1600536809008113

**Published:** 2009-03-14

**Authors:** Reza Kia, Hoong-Kun Fun, Hadi Kargar

**Affiliations:** a X-ray Crystallography Unit, School of Physics, Universiti Sains Malaysia, 11800 USM, Penang, Malaysia; b Department of Chemistry, School of Science, Payame Noor University (PNU), Ardakan, Yazd, Iran

## Abstract

The mol­ecule of the title compound, C_19_H_20_Br_2_N_2_, is a potential bidentate Schiff base ligand. The two benzene rings are inclined at a dihedral angle of 30.85 (8)°. An inter­esting feature of the crystal structure is a weak inter­molecular Br⋯Br [3.4752 (4) Å] inter­action which is shorter than the sum of the van der Waals radii of the Br atoms and links neighbouring mol­ecules into chains along the *c* axis. The crystal structure is further stabilized by inter­molecular C—H⋯π inter­actions.

## Related literature

For details of hydrogen-bond motifs, see: Bernstein *et al.* (1995 ▶). For related structure see, for example: Li *et al.* (2005 ▶); Bomfim *et al.* (2005 ▶); Glidewell *et al.* (2005 ▶, 2006 ▶); Sun *et al.* (2004 ▶); Fun *et al.* (2008 ▶). For the stability of the temperature controller used for the data collection, see: Cosier & Glazer (1986 ▶).
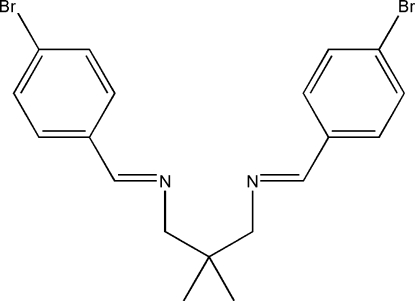


## Experimental

### 

#### Crystal data

C_19_H_20_Br_2_N_2_
*M* *_r_* = 436.19
Orthorhombic, 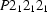

*a* = 5.6687 (1) Å
*b* = 7.7919 (2) Å
*c* = 41.5932 (9) Å
*V* = 1837.17 (7) Å^3^
*Z* = 4
Mo *K*α radiation
μ = 4.41 mm^−1^
*T* = 100 K
0.45 × 0.44 × 0.12 mm

#### Data collection

Bruker SMART APEXII CCD area-detector diffractometer
Absorption correction: multi-scan (**SADABS**; Bruker, 2005 ▶) *T*_min_ = 0.229, *T*_max_ = 0.586
38732 measured reflections
9454 independent reflections
7585 reflections with *I* > 2σ(*I*)
*R*_int_ = 0.049

#### Refinement

*R*[*F* ^2^ > 2σ(*F* ^2^)] = 0.037
*wR*(*F* ^2^) = 0.079
*S* = 1.03
9454 reflections
208 parameters
H-atom parameters constrained
Δρ_max_ = 1.04 e Å^−3^
Δρ_min_ = −0.61 e Å^−3^
Absolute structure: Flack (1983 ▶), 3971 Friedel pairs
Flack parameter: 0.019 (6)

### 

Data collection: *APEX2* (Bruker, 2005 ▶); cell refinement: *SAINT* (Bruker, 2005 ▶); data reduction: *SAINT*; program(s) used to solve structure: *SHELXTL* (Sheldrick, 2008 ▶); program(s) used to refine structure: *SHELXTL*; molecular graphics: *SHELXTL*; software used to prepare material for publication: *SHELXTL* and *PLATON* (Spek, 2009 ▶).

## Supplementary Material

Crystal structure: contains datablocks global, I. DOI: 10.1107/S1600536809008113/at2737sup1.cif

Structure factors: contains datablocks I. DOI: 10.1107/S1600536809008113/at2737Isup2.hkl

Additional supplementary materials:  crystallographic information; 3D view; checkCIF report

## Figures and Tables

**Table 1:** Hydrogen-bond geometry (Å, °)

*D*—H⋯*A*	*D*—H	H⋯*A*	*D*⋯*A*	*D*—H⋯*A*
C4—H4*A*⋯*Cg*1^i^	0.95	2.85	3.5630 (18)	132
C13—H13*A*⋯*Cg*2^ii^	0.95	2.74	3.4648 (18)	134

Symmetry codes: (i) 
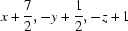
; (ii) 
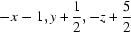
. *Cg*1 and *Cg*2 are the centroids of the C1–C6 and C9–C17 benzene rings, respectively.

**Fig. 1.**
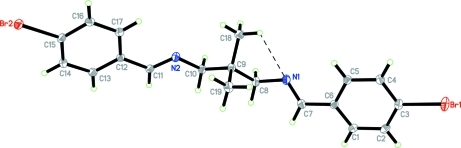
The molecular structure of the title compound with atom labels and 50% probability ellipsoids for non-H atoms. Intramolecular hydrogen bond is shown as dashed line.

**Fig. 2.**
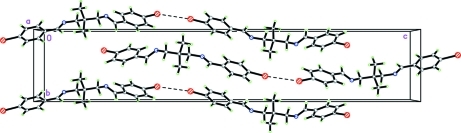
The crystal packing of the title compound, viewed down the a- axis showing chains along the c-axis by Br···Br interactions.

## supplementary crystallographic information

### Comment

Schiff bases are one of most prevalent mixed-donor ligands in the field of coordination chemistry. They play an important role in the development of coordination chemistry related to catalysis and enzymatic reactions, magnetism, and supramolecular architectures. Structures of Schiff bases derived from substituted benzaldehydes and closely related to the title compound have been reported previously (Li *et al.*, 2005; Bomfim *et al.*, 2005; Glidewell *et al.*, 2005, 2006; Sun *et al.*, 2004; Fun *et al.*, 2008).

In the title compound, Fig. 1, intramolecular C—H···N hydrogen bonds forms five-membered rings, producing *S*(5) ring motifs (Bernstein *et al.*, 1995). The two benzene rings make a dihedral angle of 30.85 (8)°. The crystal structure is further stabilized by weak intermolecular C—H···π interactions [*Cg*1 and *Cg*2 are the centroids of the C1–C6 and C12–C17 benzene rings] (Table 1). The interesting feature of the crystal structure is weak intermolecular Br···Br [3.4752 (4) Å; symmetry code: 5/2 - *x*, 1 - *y*, -1/2 + *z*] interaction which is shorter than the sum of the van der Waals radius of Br atoms and link neighbouring molecules into chains along the *c* axis (Fig. 2).

### Experimental

The synthetic method has been described earlier (Fun *et al.*, 2008), except that 4-bromobenzaldehyde was used. Single crystals suitable for *X*-ray diffraction were obtained by evaporation of an ethanol solution at room temperature.

### Refinement

All of the hydrogen atoms were positioned geometrically and refined using a riding model approximation with C—H = 0.95–0.99 Å and *U*_iso_(H) = 1.2 or 1.5*U*_eq_(C). In the presence of the sufficient anomalous scattering, the absoulte configuration was determined (3971 Friedel pairs).

### Crystal data

**Table:**

C_19_H_20_Br_2_N_2_	*F*(000) = 872
*M**_r_* = 436.19	*D*_x_ = 1.577 Mg m^−^^3^
Orthorhombic, *P*2_1_2_1_2_1_	Mo *K*α radiation, λ = 0.71073 Å
Hall symbol: P 2ac 2ab	Cell parameters from 9987 reflections
*a* = 5.6687 (1) Å	θ = 2.7–35.5°
*b* = 7.7919 (2) Å	µ = 4.41 mm^−^^1^
*c* = 41.5932 (9) Å	*T* = 100 K
*V* = 1837.17 (7) Å^3^	Block, colourless
*Z* = 4	0.45 × 0.44 × 0.12 mm

### Data collection

**Table:**

Bruker SMART APEXII CCD area-detector diffractometer	9454 independent reflections
Radiation source: fine-focus sealed tube	7585 reflections with *I* > 2σ(*I*)
graphite	*R*_int_ = 0.049
φ and ω scans	θ_max_ = 37.5°, θ_min_ = 2.0°
Absorption correction: multi-scan (*SADABS*; Bruker, 2005)	*h* = −9→9
*T*_min_ = 0.229, *T*_max_ = 0.586	*k* = −10→13
38732 measured reflections	*l* = −71→55

### Refinement

**Table:**

Refinement on *F*^2^	Secondary atom site location: difference Fourier map
Least-squares matrix: full	Hydrogen site location: inferred from neighbouring sites
*R*[*F*^2^ > 2σ(*F*^2^)] = 0.037	H-atom parameters constrained
*wR*(*F*^2^) = 0.079	*w* = 1/[σ^2^(*F*_o_^2^) + (0.0316*P*)^2^ + 0.2032*P*] where *P* = (*F*_o_^2^ + 2*F*_c_^2^)/3
*S* = 1.03	(Δ/σ)_max_ = 0.011
9454 reflections	Δρ_max_ = 1.04 e Å^−^^3^
208 parameters	Δρ_min_ = −0.60 e Å^−^^3^
0 restraints	Absolute structure: Flack (1983), 3971 Friedel pairs
Primary atom site location: structure-invariant direct methods	Flack parameter: 0.019 (6)

### Special details

**Table:**

Experimental. The crystal was placed in the cold stream of an Oxford Cyrosystems Cobra open-flow nitrogen cryostat (Cosier & Glazer, 1986) operating at 100.0 (1) K.
Geometry. All e.s.d.'s (except the e.s.d. in the dihedral angle between two l.s. planes) are estimated using the full covariance matrix. The cell e.s.d.'s are taken into account individually in the estimation of e.s.d.'s in distances, angles and torsion angles; correlations between e.s.d.'s in cell parameters are only used when they are defined by crystal symmetry. An approximate (isotropic) treatment of cell e.s.d.'s is used for estimating e.s.d.'s involving l.s. planes.
Refinement. Refinement of *F*^2^ against ALL reflections. The weighted *R*-factor *wR* and goodness of fit *S* are based on *F*^2^, conventional *R*-factors *R* are based on *F*, with *F* set to zero for negative *F*^2^. The threshold expression of *F*^2^ > σ(*F*^2^) is used only for calculating *R*-factors(gt) *etc*. and is not relevant to the choice of reflections for refinement. *R*-factors based on *F*^2^ are statistically about twice as large as those based on *F*, and *R*- factors based on ALL data will be even larger.

### Fractional atomic coordinates and isotropic or equivalent isotropic displacement parameters (Å^2^)

**Table:**

	*x*	*y*	*z*	*U*_iso_*/*U*_eq_	
Br1	1.59060 (4)	0.70918 (3)	0.679860 (5)	0.03041 (5)	
Br2	1.02132 (3)	0.35735 (2)	1.095005 (4)	0.02216 (4)	
N1	0.9734 (3)	0.7039 (2)	0.82239 (4)	0.0214 (3)	
N2	0.8180 (3)	0.6175 (2)	0.93830 (4)	0.0198 (3)	
C1	1.0509 (3)	0.5599 (2)	0.74067 (5)	0.0203 (3)	
H1A	0.9062	0.4991	0.7388	0.024*	
C2	1.1894 (4)	0.5835 (3)	0.71353 (5)	0.0219 (3)	
H2A	1.1410	0.5403	0.6932	0.026*	
C3	1.4013 (3)	0.6720 (2)	0.71684 (4)	0.0212 (3)	
C4	1.4767 (3)	0.7348 (2)	0.74652 (4)	0.0197 (3)	
H4A	1.6230	0.7935	0.7484	0.024*	
C5	1.3353 (3)	0.7104 (3)	0.77327 (4)	0.0184 (3)	
H5A	1.3848	0.7528	0.7936	0.022*	
C6	1.1191 (3)	0.6235 (2)	0.77059 (4)	0.0180 (3)	
C7	0.9574 (3)	0.6067 (2)	0.79820 (5)	0.0198 (3)	
H7A	0.8386	0.5208	0.7978	0.024*	
C8	0.8001 (3)	0.6814 (3)	0.84805 (4)	0.0209 (3)	
H8A	0.6996	0.5808	0.8432	0.025*	
H8B	0.6972	0.7839	0.8491	0.025*	
C9	0.9225 (3)	0.6548 (2)	0.88094 (4)	0.0179 (3)	
C10	0.7242 (3)	0.6457 (2)	0.90606 (4)	0.0188 (3)	
H10A	0.6333	0.7542	0.9057	0.023*	
H10B	0.6152	0.5510	0.9005	0.023*	
C11	0.6955 (3)	0.5293 (2)	0.95790 (4)	0.0178 (3)	
H11A	0.5486	0.4841	0.9509	0.021*	
C12	0.7732 (3)	0.4951 (2)	0.99097 (4)	0.0165 (3)	
C13	0.6324 (3)	0.3938 (2)	1.01110 (5)	0.0184 (3)	
H13A	0.4861	0.3514	1.0033	0.022*	
C14	0.7025 (3)	0.3543 (3)	1.04224 (4)	0.0196 (3)	
H14A	0.6050	0.2867	1.0558	0.024*	
C15	0.9177 (3)	0.4158 (2)	1.05301 (4)	0.0184 (3)	
C16	1.0606 (3)	0.5189 (2)	1.03375 (4)	0.0192 (3)	
H16A	1.2063	0.5616	1.0417	0.023*	
C17	0.9877 (3)	0.5583 (2)	1.00290 (4)	0.0193 (3)	
H17A	1.0838	0.6289	0.9897	0.023*	
C18	1.0856 (3)	0.8053 (3)	0.88844 (5)	0.0241 (4)	
H18A	1.1615	0.7866	0.9093	0.036*	
H18B	0.9935	0.9117	0.8891	0.036*	
H18C	1.2065	0.8142	0.8717	0.036*	
C19	1.0619 (4)	0.4868 (3)	0.88061 (5)	0.0244 (4)	
H19A	1.1393	0.4706	0.9015	0.037*	
H19B	1.1816	0.4913	0.8636	0.037*	
H19C	0.9544	0.3908	0.8765	0.037*	

### Atomic displacement parameters (Å^2^)

**Table:**

	*U*^11^	*U*^22^	*U*^33^	*U*^12^	*U*^13^	*U*^23^
Br1	0.02919 (10)	0.04538 (12)	0.01667 (8)	0.00266 (9)	0.00467 (7)	0.00429 (9)
Br2	0.02804 (9)	0.02117 (7)	0.01728 (8)	−0.00042 (7)	−0.00430 (7)	0.00085 (7)
N1	0.0223 (6)	0.0248 (7)	0.0169 (6)	−0.0017 (6)	0.0014 (6)	0.0021 (6)
N2	0.0204 (7)	0.0234 (8)	0.0155 (7)	0.0001 (6)	0.0011 (5)	0.0003 (6)
C1	0.0215 (8)	0.0207 (8)	0.0189 (8)	0.0001 (6)	−0.0015 (6)	−0.0033 (6)
C2	0.0239 (8)	0.0251 (8)	0.0168 (8)	0.0028 (7)	−0.0041 (7)	−0.0041 (7)
C3	0.0228 (7)	0.0233 (9)	0.0176 (8)	0.0044 (6)	0.0029 (6)	0.0023 (6)
C4	0.0183 (7)	0.0218 (7)	0.0190 (7)	−0.0006 (6)	0.0004 (6)	0.0019 (6)
C5	0.0194 (7)	0.0208 (8)	0.0150 (7)	−0.0003 (7)	−0.0011 (6)	−0.0011 (7)
C6	0.0209 (7)	0.0170 (8)	0.0161 (7)	0.0012 (6)	0.0000 (6)	0.0006 (6)
C7	0.0178 (7)	0.0216 (8)	0.0199 (8)	−0.0008 (6)	−0.0005 (6)	0.0034 (6)
C8	0.0181 (7)	0.0277 (10)	0.0168 (8)	−0.0001 (6)	0.0012 (6)	0.0024 (7)
C9	0.0160 (7)	0.0201 (7)	0.0175 (7)	0.0003 (6)	0.0006 (5)	0.0009 (6)
C10	0.0186 (7)	0.0221 (7)	0.0157 (7)	−0.0007 (6)	−0.0008 (6)	0.0019 (8)
C11	0.0191 (7)	0.0186 (8)	0.0157 (7)	−0.0003 (6)	0.0002 (6)	−0.0017 (6)
C12	0.0181 (7)	0.0161 (7)	0.0152 (7)	0.0006 (6)	0.0006 (6)	−0.0013 (6)
C13	0.0166 (7)	0.0197 (8)	0.0189 (8)	−0.0009 (6)	0.0002 (6)	−0.0003 (6)
C14	0.0206 (7)	0.0205 (7)	0.0178 (8)	−0.0013 (7)	0.0020 (6)	0.0015 (7)
C15	0.0226 (8)	0.0186 (7)	0.0141 (7)	0.0027 (6)	−0.0013 (6)	−0.0020 (6)
C16	0.0176 (8)	0.0191 (7)	0.0208 (8)	−0.0016 (6)	0.0004 (6)	−0.0026 (6)
C17	0.0206 (7)	0.0192 (7)	0.0180 (7)	−0.0022 (7)	0.0004 (7)	−0.0009 (6)
C18	0.0212 (8)	0.0259 (9)	0.0251 (9)	−0.0045 (7)	0.0019 (7)	−0.0011 (8)
C19	0.0246 (9)	0.0235 (8)	0.0250 (9)	0.0057 (7)	0.0037 (7)	0.0021 (7)

### Geometric parameters (Å, °)

**Table:**

Br1—C3	1.8978 (19)	C9—C19	1.529 (3)
Br2—C15	1.8983 (18)	C9—C10	1.536 (2)
N1—C7	1.263 (2)	C10—H10A	0.9900
N1—C8	1.462 (2)	C10—H10B	0.9900
N2—C11	1.272 (2)	C11—C12	1.469 (3)
N2—C10	1.459 (2)	C11—H11A	0.9500
C1—C2	1.387 (3)	C12—C13	1.400 (3)
C1—C6	1.394 (3)	C12—C17	1.402 (3)
C1—H1A	0.9500	C13—C14	1.389 (3)
C2—C3	1.392 (3)	C13—H13A	0.9500
C2—H2A	0.9500	C14—C15	1.385 (3)
C3—C4	1.395 (3)	C14—H14A	0.9500
C4—C5	1.384 (2)	C15—C16	1.394 (3)
C4—H4A	0.9500	C16—C17	1.383 (3)
C5—C6	1.404 (3)	C16—H16A	0.9500
C5—H5A	0.9500	C17—H17A	0.9500
C6—C7	1.475 (3)	C18—H18A	0.9800
C7—H7A	0.9500	C18—H18B	0.9800
C8—C9	1.548 (3)	C18—H18C	0.9800
C8—H8A	0.9900	C19—H19A	0.9800
C8—H8B	0.9900	C19—H19B	0.9800
C9—C18	1.525 (3)	C19—H19C	0.9800
			
C7—N1—C8	117.50 (17)	C9—C10—H10A	109.3
C11—N2—C10	118.11 (16)	N2—C10—H10B	109.3
C2—C1—C6	121.48 (17)	C9—C10—H10B	109.3
C2—C1—H1A	119.3	H10A—C10—H10B	108.0
C6—C1—H1A	119.3	N2—C11—C12	122.30 (17)
C1—C2—C3	118.26 (18)	N2—C11—H11A	118.9
C1—C2—H2A	120.9	C12—C11—H11A	118.9
C3—C2—H2A	120.9	C13—C12—C17	118.72 (17)
C2—C3—C4	121.73 (18)	C13—C12—C11	119.43 (16)
C2—C3—Br1	118.89 (15)	C17—C12—C11	121.84 (17)
C4—C3—Br1	119.37 (14)	C14—C13—C12	121.29 (17)
C5—C4—C3	119.03 (17)	C14—C13—H13A	119.4
C5—C4—H4A	120.5	C12—C13—H13A	119.4
C3—C4—H4A	120.5	C15—C14—C13	118.47 (17)
C4—C5—C6	120.53 (16)	C15—C14—H14A	120.8
C4—C5—H5A	119.7	C13—C14—H14A	120.8
C6—C5—H5A	119.7	C14—C15—C16	121.70 (17)
C1—C6—C5	118.96 (16)	C14—C15—Br2	119.15 (14)
C1—C6—C7	119.40 (17)	C16—C15—Br2	119.15 (14)
C5—C6—C7	121.56 (16)	C17—C16—C15	119.17 (17)
N1—C7—C6	121.50 (17)	C17—C16—H16A	120.4
N1—C7—H7A	119.3	C15—C16—H16A	120.4
C6—C7—H7A	119.3	C16—C17—C12	120.63 (17)
N1—C8—C9	111.10 (15)	C16—C17—H17A	119.7
N1—C8—H8A	109.4	C12—C17—H17A	119.7
C9—C8—H8A	109.4	C9—C18—H18A	109.5
N1—C8—H8B	109.4	C9—C18—H18B	109.5
C9—C8—H8B	109.4	H18A—C18—H18B	109.5
H8A—C8—H8B	108.0	C9—C18—H18C	109.5
C18—C9—C19	110.28 (15)	H18A—C18—H18C	109.5
C18—C9—C10	109.88 (15)	H18B—C18—H18C	109.5
C19—C9—C10	110.16 (15)	C9—C19—H19A	109.5
C18—C9—C8	110.46 (16)	C9—C19—H19B	109.5
C19—C9—C8	109.78 (16)	H19A—C19—H19B	109.5
C10—C9—C8	106.20 (14)	C9—C19—H19C	109.5
N2—C10—C9	111.43 (14)	H19A—C19—H19C	109.5
N2—C10—H10A	109.3	H19B—C19—H19C	109.5
			
C6—C1—C2—C3	0.4 (3)	C11—N2—C10—C9	−146.93 (17)
C1—C2—C3—C4	0.7 (3)	C18—C9—C10—N2	−61.5 (2)
C1—C2—C3—Br1	−178.88 (14)	C19—C9—C10—N2	60.2 (2)
C2—C3—C4—C5	−0.9 (3)	C8—C9—C10—N2	178.99 (16)
Br1—C3—C4—C5	178.64 (14)	C10—N2—C11—C12	−179.41 (16)
C3—C4—C5—C6	0.1 (3)	N2—C11—C12—C13	−178.58 (17)
C2—C1—C6—C5	−1.2 (3)	N2—C11—C12—C17	0.2 (3)
C2—C1—C6—C7	175.36 (17)	C17—C12—C13—C14	−0.6 (3)
C4—C5—C6—C1	0.9 (3)	C11—C12—C13—C14	178.16 (17)
C4—C5—C6—C7	−175.54 (17)	C12—C13—C14—C15	−0.8 (3)
C8—N1—C7—C6	177.89 (16)	C13—C14—C15—C16	1.7 (3)
C1—C6—C7—N1	−157.81 (18)	C13—C14—C15—Br2	−178.06 (14)
C5—C6—C7—N1	18.7 (3)	C14—C15—C16—C17	−1.2 (3)
C7—N1—C8—C9	126.29 (18)	Br2—C15—C16—C17	178.54 (14)
N1—C8—C9—C18	57.0 (2)	C15—C16—C17—C12	−0.2 (3)
N1—C8—C9—C19	−64.8 (2)	C13—C12—C17—C16	1.1 (3)
N1—C8—C9—C10	176.10 (15)	C11—C12—C17—C16	−177.63 (17)

### Hydrogen-bond geometry (Å, °)

**Table:**

*D*—H···*A*	*D*—H	H···*A*	*D*···*A*	*D*—H···*A*
C18—H18C···N1	0.98	2.59	2.929 (3)	101
C4—H4A···Cg1^i^	0.95	2.85	3.5630 (18)	132
C13—H13A···Cg2^ii^	0.95	2.74	3.4648 (18)	134

Symmetry codes: (i) *x*+7/2, −*y*+1/2, −*z*+1; (ii) −*x*−1, *y*+1/2, −*z*+5/2.
